# Montreal Cognitive Assessment for Evaluating Cognitive Impairment in Subarachnoid Hemorrhage: A Systematic Review

**DOI:** 10.3390/jcm11164679

**Published:** 2022-08-10

**Authors:** Amalia Cornea, Mihaela Simu, Elena Cecilia Rosca

**Affiliations:** 1 Department of Neurology, Victor Babes University of Medicine and Pharmacy Timisoara, Eftimie Murgu Square No. 2, 300041 Timisoara, Romania; 2 Department of Neurology, Clinical Emergency County Hospital Timisoara, Boulevard Iosif Bulbuca No. 10, 300736 Timisoara, Romania

**Keywords:** Montreal Cognitive Assessment, MoCA, subarachnoid hemorrhage, systematic review

## Abstract

Subarachnoid hemorrhage (SAH) is a severe condition with high mortality and extensive long-term morbidity. Although research has focused mainly on physical signs and disability for decades, in recent years, it has been increasingly recognized that cognitive and psychological impairments may be present in many patients with SAH, negatively impacting their quality of life. We performed a systematic review aiming to provide a comprehensive report on the diagnostic accuracy of the Montreal Cognitive Assessment (MoCA) test for evaluating the presence of cognitive impairment in patients with SAH. Using appropriate search terms, we searched five databases (PubMed, Scopus, PsychINFO, Web of Sciences, and Latin American and Caribbean Health Sciences Literature) up to January 2022. Two cross-sectional studies investigated the accuracy of MoCA in SAH patients in the subacute and chronic phase. We appraised the quality of the included studies using the Quality Assessment of Diagnostic Accuracy Studies 2 (QUADAS-2) criteria. The MoCA test provides information about general cognitive functioning disturbances. However, a lower threshold than the original cutoff might be needed as it improves diagnostic accuracy, lowering the false positive rates. Further research is necessary for an evidence-based decision to use the MoCA in SAH patients.

## 1. Introduction

Subarachnoid hemorrhage (SAH) is a severe condition resulting from blood accumulation between the arachnoid and pia mater. The acute bleeding into the subarachnoid space may have multiple causes, the most frequent being the nontraumatic spontaneous subarachnoid hemorrhage. In adults, most of the primary SAH is due to the rupture of an intracerebral aneurysm; in children, the majority of SAH is due to bleeding of a cerebral arteriovenous malformation. Nonetheless, some patients with primary SAH may present no evidence of cerebral aneurism or other vascular malformations (non-aneurysmal SAH) in approximately 10% of cases (peri-mesencephalic SAH) [1,2,3].

Secondary SAH’s etiology comprises trauma, reversible cerebral vasoconstriction syndrome, posterior reversible encephalopathy syndrome (PRES), cerebral amyloid angiopathy, cerebral vasculitis, and cerebral venous sinus thrombosis coagulopathies, tumors, drugs, septic emboli from endocarditis, and iatrogenic factors [1,3].

Despite it being rare for it to be the cause of stroke, accounting for 1–6% of all strokes [4], patients with SAH have high mortality and extensive long-term morbidity [4,5].

Adequate management of SAH is essential, and the guidelines provide several key recommendations, including different aspects related to SAH complications [5,6].

Among complications, patients may present cognitive impairment. Although research has focused mainly on physical signs and disability for decades, it is increasingly recognized that cognitive and psychological impairments may be present in many patients with SAH, negatively impacting their quality of life [7,8,9]. Consequently, in 2019, an international, multidisciplinary ad-hoc panel of experts in clinical outcomes proposed several recommendations on over 50 outcome measures after SAH. Among them, the modified Rankin Scale (mRS) score and the Montreal Cognitive Assessment (MoCA) test were considered preferred outcomes and classified as “Supplemental—Highly Recommended” [7].

The MoCA is a brief cognitive test developed in 2005 to detect mild cognitive impairment (MCI). Initially, it was reported to present high sensitivity and specificity in the older adult population [10]. Later, various studies reported that the test presented good psychometric properties, with good sensitivity in identifying mild cognitive impairment in several neurological conditions. For example, in patients with MCI, the MoCA presented excellent internal consistency, the Cronbach’s alpha being 0.83 on the standardized items [10]. It also has a good test-retest reliability, with a mean change in the scores from the first to the second evaluation of 0.9 points [10]. Moreover, studies using Rasch analysis techniques indicated that the MoCA scores could quantify the cognitive ability of an individual, successfully tracking the changes in cognitive functioning over time [11]. Hence, the MoCA has widespread international use. It is available in nearly 100 languages and is considered one of the best cognitive screening tools [12].

The MoCA is validated for different neurological disorders, like MCI, Alzheimer’s disease [13], and Parkinson’s disease [14]. However, recent systematic reviews and meta-analyses found that the usual threshold of 26 may not offer the best tradeoff between sensitivity and specificity. The optimal threshold was reported to be 22 in stroke patients [15]. Furthermore, another systematic review found that the cutoff of 23 was optimal for differentiating healthy cognitive aging from possible MCI, maximizing true positive cases and minimizing false-positive results [16]. In addition, thresholds lower than 26 offered a better balance between true-positive and false-positive results in patients with Alzheimer’s dementia [17] and people living with HIV [18].

An early and correct diagnosis of cognitive impairment in patients with SAH is essential, as it is a significant cause of functional disability and related outcomes. Furthermore, various rehabilitation strategies (speech, occupational, and cognitive therapy) might improve cognitive functions, and a personalized approach would benefit SAH patients.

Therefore, investigating and validating tests that measure cognitive functions are critical to better treating the cognitive impairment in SAH patients. In addition, the neuropsychological assessment has a pivotal role in identifying cognitive changes early in the disease, monitoring progression, and evaluating the outcome of therapeutic interventions.

The MoCA test achieves critical feasibility criteria for use in clinical practice The administration time is short (10 min), and multiple translations are available Moreover, online training and certification can be obtained on the MoCA website. The test evaluates a broad range of cognitive domains and was demonstrated to present good psychometric properties in other neurological diseases. Hence, it may help identify patients with cognitive dysfunction that might require further evaluations and specific care, enabling access to appropriate services. However, a false-positive result may imply high costs due to additional unneeded investigations.

Consequently, there is a significant value in reviewing the empirical research that supports the use of MoCA as a screening tool for cognitive impairment in SAH patients.

We aimed to provide a comprehensive review of existing literature by investigating the evidence on using the MoCA test in SAH and to lay out a basis for rational decision-making, emphasizing possible answers that are easily accessible to clinicians, health care professionals, and policymakers. We also aimed to indicate research gaps that require attention.

## 2. Materials and Methods

The present systematic review was performed following the recommendations described in the Cochrane Handbook for Diagnostic Test Accuracy Reviews [19] and the guidelines of the Preferred Reporting Items for Systematic Reviews and Meta-Analysis (PRISMA) for Diagnostic Test Accuracy [20] (see Supplementary Material S1).

### 2.1. Research Questions

Our objective was to systematically review the research regarding the accuracy of MoCA in diagnosing cognitive impairment in patients with SAH and the quantity of evidence available on its use. Moreover, we aimed to assess the methodological quality (in terms of risk of bias) of studies on this topic and to identify research gaps concerning this screening test.

### 2.2. Search Strategy and Eligible Studies

We performed a computerized bibliographic search from inception to 16 January 2022 in the following databases: PubMed, Scopus, Web of Sciences, PsychINFO, and Latin American and Caribbean Health Sciences Literature (LILACS). To develop a comprehensive search strategy, we used search strings that refer to the index test and the target condition using the following keywords: “Montreal Cognitive Assessment” OR “MoCA” AND “subarachnoid hemorrhage” [MeSH]. These search terms were for PubMed, the primary source of citations. Searches in other data sources used similar versions of these terms as appropriate for each database. We did not use other search filters because we aimed to generate a broad list of research. In addition, a manual search was performed on the MoCA website and by checking reference lists of all relevant articles to identify possible additional studies. We did not apply any language restrictions to our searches.

### 2.3. Study Selection

Two authors reviewed the title, abstract, and full text (when needed) of all retrieved records and evaluated whether the study met the inclusion criteria. Any article that was considered eligible by either reviewer in the abstract stage was assessed in full text. We solved disagreements through discussions; the participation of a third rater was not necessary to address discrepancies.

To systematically review the literature on the use of MoCA in the context of SAH, we selected all the studies where MoCA was used to assess the cognitive status of SAH patients. The main types of eligible studies were: (i) cross-sectional studies in which participants received the index test (MoCA) and a reference standard diagnostic assessment; (ii) case-control studies that compare MoCA to a battery of tests; and (iii) studies comparing MoCA to another short cognitive test (e.g., MMSE).

We included prospective or retrospective, observational or interventional studies, where MoCA was used to assess the cognitive functions in SAH patients and compared to a reference standard. Intervention studies were not excluded in the abstract stage because the data on diagnostic test accuracy may be present in studies that do not have, as a primary objective, a test accuracy estimation. If available, besides primary studies, we also intended to include systematic reviews.

We selected studies reporting adults (over 18 years old) with confirmed SAH. The index test was any full version of MoCA. We expected to find the recommended threshold of 26 or below to differentiate normal (≥26) from impaired cognition (<26); however, we also planned to include studies using other cutoff scores. The target condition was cognitive impairment, including MCI and dementia. We used as a reference standard for cognitive dysfunction a complex neuropsychological evaluation, assessing at least five neurocognitive domains (including verbal and language skills, attention and working memory, learning and recall, abstraction and executive functions, speed of information processing, and motor skills), with endorsed recommendations on appropriate tests.

Studies with less than 10 participants were excluded. In addition, we did not include studies with patients with confounding factors such as neurological disorders (e.g., recent traumatic brain injury, CNS infections, other types of strokes, other neurodegenerative disorders, and brain tumors), drug or alcohol addiction, and active infections.

### 2.4. Data Extraction

We present the review findings in a tabular form to provide a descriptive summary of the results. We collected the following essential information: the source of the data (e.g., author, year of publication, where the study was conducted), methods of the research, and the data relevant to our review questions. The latter includes the number of patients, age, gender, education, type of SAH, time from onset, scores on MoCA, scores on other cognitive tests, and functional status.

Data were extracted independently by two authors; a third reviewer solved any discrepancies.

### 2.5. Quality Assessment

The methodological quality of the included studies was assessed by two authors independently, using the unmodified Quality Assessment of Diagnostic Accuracy Studies 2 tool [21]. All disagreements were solved through discussions.

## 3. Results

Our search strategy revealed 478 results. From a total of 95 unique studies identified and assessed in the full text, we included two cross-sectional studies comparing the MoCA to a battery of tests.

The list of excluded studies with reasons for exclusion is presented in Supplementary Material S2.

**Figure 1:**
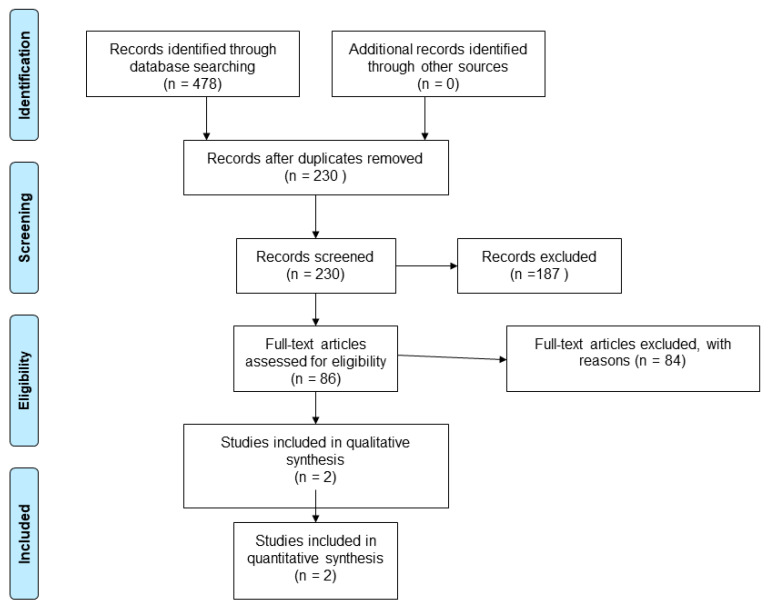
Flow diagram showing the process for inclusion of studies assessing the diagnostic test accuracy of MoCA in SAH patients.

The characteristics of the included studies are presented in Table 1. The PRISMA diagram reporting the selection process of studies is detailed in Figure 1.

The year of publication was 2012 [22] and 2013 [23]. The study samples were selected from two different countries (Canada and China). Samples varied in size (32–80 participants), sex ratio, median age (55.2–58 years), MoCA scores, and functional status. The characteristics of the included studies are presented in Table 1.

To date, only two studies have assessed the validity of the MoCA as a screening tool for cognitive impairment in SAH, using an extensive cognitive battery as a reference standard.

The study of Schweizer et al. [22] investigated 32 individuals with aneurismal SAH who had made a good recovery. The authors included a highly selected population, with patients only with a good outcome (*n* = 31) or moderate disability (*n =* 1) on the Glasgow Outcome Scale (GOS). At the time of evaluation, none of the patients presented evidence of significant neurological deficits like paresis or plegia [22]. The neurocognitive evaluation was performed at least six months after the SAH. The reference standard investigated attention (Trail Making Test A [TMT-A], omission errors on the Sustained Attention to Response Test [SART]), executive functions (Wisconsin Card Sorting Test [WCST], Trail Making Test B [TMT-B], commission errors on the SART), verbal learning and memory (California Verbal Learning Test [CVLT]), language skills (Boston Naming Test [BNT]), and motor functions (Grooved Pegboard [GP]) [22]. The MoCA scores (25.4 ± 2.8) were considerably lower than the MMSE scores (29.3 ± 1.1) (*p* < 0.001). Using the recommended threshold of 26, 42% of patients presented cognitive impairment on the MoCA test; however, with the usual cutoffs, 0% of cases were found to be cognitively impaired on the MMSE test [22]. The frontal lobe functions were most commonly affected (e.g., executive functions, attention, language, and motor skills). 

The authors compared the MoCA scores with each test used for the reference standard. The sensitivity of MoCA ranged from 0.40 to 1.00; the highest sensitivity was reported when compared to the BNT (0.86) and the CVLT Trials 1–5 (1.00). The specificity of the MoCA was moderate (0.54–0.70) for all reference tests [22].

The study of Wong et al. [23] investigated the cognitive functions in SAH patients with spontaneous aneurismal SAH aged between 21 and 75 years. The authors administered MoCA and MMSE 2–4 weeks (subacute phase) and one year (chronic phase) after the stroke. The cutoff for mild cognitive impairment was 24/25 for MoCA. The Cantonese version of the MMSE was validated in a population of individuals with dementia, for whom the optimal cutoff was found to be 19/20 [23]. The reference standard included a battery that was validated for Chinese patients, assessing verbal memory (Hong Kong List Learning Test [HKLLT]), visuospatial skills and memory (Rey Osterrieth Complex Figure Test), attention and working memory (verbal and visual digit span forward and backward subtests from the Chinese Wechsler Memory Scale), executive functions and psychomotor speed (Symbol-Digit Modalities Test, Color Trails Test [CTT], Animal fluency), and language (modified Boston Naming Test [mBNT]) [23].

The MoCA and MMSE presented similar AUCs in the subacute phase (2–4 weeks). In the chronic phase (1 year), the MoCA presented significantly higher AUCs than the MMSE for cognitive impairment. The authors defined cognitive impairment as deficits in two or more cognitive domains. The optimal cutoff for MoCA was ≤18 at 2–4 weeks after SAH and ≤22 at one year. For the MMSE, the optimal threshold was 24 in both the subacute and chronic phases. The diagnostic accuracy ranged from 80% to 92%.

For the MoCA test, the cutoff of 17/18 provided a sensitivity of 0.75 (95%CI 0.43–0.95), a specificity of 0.95 (95%CI 87–99), with a positive predictive value (PPV) of 0.75 (41–95), and a negative predictive value (NPV) of 0.95 (95%CI 87–99). The accuracy was 92% [23]. At one year, the MoCA cutoff score of 21/22 presented a sensitivity of 1.0 (95%CI 74–100), a specificity of 0.75 (95%CI 63–85), a PPV of 0.41 (95%CI 24–61), and an NPV of 1.00 (95%CI 93–100). The diagnostic accuracy was 85% [23].

Regarding the risk of bias, we found several methodological problems. In the patient’s spectrum domain of the included studies, the sampling method for inclusion may lead to important variations in diagnostic accuracy. Ideally, the authors should prospectively include a consecutive or random series of individuals fulfilling all the selection criteria. The risk of introducing bias into the study is high if other sampling methods are used [19]. Schweizer et al. [22] excluded severe SAH cases when recruiting a consecutive sample of patients. Wong et al. [23] did not report the methods used to sample the SAH patients.

In the index test domain, both studies were considered to present an unclear risk of bias; the authors did not specify whether they interpreted the results of the index test without knowledge of the reference standard scores. None of the studies provided data on the blinding of assessors with regard to the reference standard (diagnostic review bias). Research demonstrated that both types of bias (test review and diagnostic review) would increase the sensitivity of the index test. However, no systematic effect on specificity was observed [24].

The period between the administration of MoCA and the reference standard was adequate in one study [23] and unclear in the other study [22]. The index test and the reference standard battery should be administered at the same study visit or in a short time frame. Otherwise, misclassification due to recovery, benefit from treatment, progression to a more advanced stage, or occurrence of a new disease may interfere with the results if a delay occurs [24].

**Figure 2:**
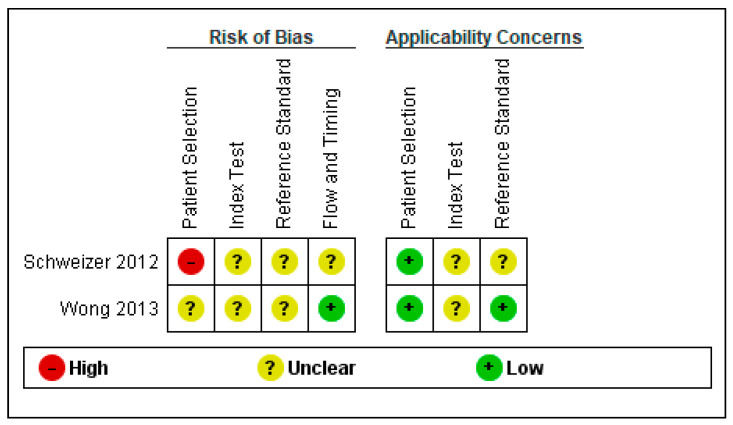
Risk of bias and applicability concerns graph: review authors’ judgments about each domain presented as percentages across included studies [22,23].

**Figure 3:**
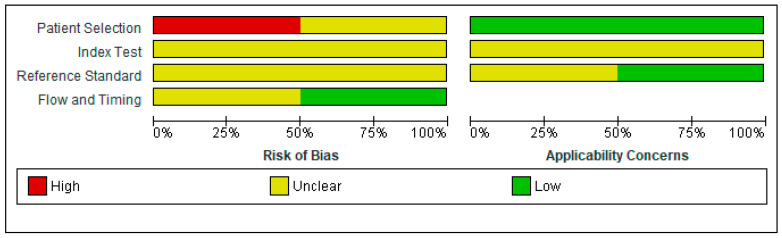
Risk of bias and applicability concerns summary: review authors’ judgments about each domain for each included study.

The QUADAS-2scores for each domain are presented in Figure 2 and Figure 3.

## 4. Discussion

The present systematic review found only two studies on the diagnostic accuracy of MoCA in SAH patients. The researchers report a fair accuracy of the MoCA in diagnosing post-aneurismal SAH at both subacute and chronic stages. However, the optimal thresholds for subacute and chronic patients were lower than the recommended cutoff of 26 [23]. Furthermore, the MoCA was found to be superior to the MMSE in this specific pathology [22,23].

The greater sensitivity reported on MoCA could be explained by the fact that the test assesses several cognitive domains that the MMSE does not. For example, MoCA includes subtests that measure executive function and abstraction, tasks that are frequently affected after SAH [25]. Moreover, some subtests are more complex than MMSE items (e.g., memory, visuospatial skills, language). The lower sensitivity of MMSE can be attributed to a ceiling effect, as all the SAH patients scored ≥27 on the MMSE [22]. Previous studies reported a similar ceiling effect on patients with MCI [10]. Nonetheless, the MoCA had lower specificity than the MMSE, suggesting that, in some patients, the MoCA may present higher rates of false-positive results. These findings align with other studies on patients with ischemic stroke [26,27], or MCI [28,29].

In both studies, the reference standard was composed of several tests assessing multiple cognitive domains. Studies on the use of complex neuropsychological batteries in healthy adults report that 15–22% of individuals from a normal control group and 20% of a simulated normal population will present scores below the threshold for cognitive impairment [30,31]. These false-positive errors are generated by two frequent practices aimed at increasing sensitivity in detecting the milder forms of cognitive dysfunction. The administration of composite test batteries will determine higher false-positive rates than individual tests, as they require multiple comparisons. The probability of abnormal scores increases as the number of tests performed per domain and the number of assessed cognitive domains increase. In addition, high cutoff scores (i.e., z-scores with a threshold of 1 SD) will increase the overlap between critical portions of test-score distributions in patients with and without the disease [30,31]. Therefore, aiming for an increased sensitivity will determine a reduction of the specificity. As a consequence, the false-positive cases will bias the prevalence estimates and will determine reductions in power for analytical estimates [30,31].

In addition, none of the studies provided data on the psychometric properties of MoCA in SAH patients (e.g., internal consistency, Cronbach’s alpha, test-retest, and interrater reliability). Therefore, the results of a SAH patient on the MoCA test must be interpreted with caution. Despite the current international recommendations, the data regarding its diagnostic accuracy and use are limited [7]. The use of this brief cognitive screening tool requires additional, extensive testing for complete validation and to determine the severity of the cognitive impairment.

Although the MoCA test is a promising screening tool for patients with SAH, our systematic review found that further studies are necessary regarding its diagnostic accuracy and use. Even if it demonstrated good sensitivity and specificity, the optimal cutoff is unclear. In patients with other neurological disorders, it was reported that a lower threshold offers a better balance between true-positive and false-positive results [15,16,17,18,32]. Consequently, further studies are necessary to investigate the optimal cutoff.

The present systematic review reaffirms the main potential advantage of the MoCA as a screening test promising to decrease neuropsychological assessment time and costs significantly. However, different thresholds should be investigated in different languages for individuals with multiple educational and cultural backgrounds. Researchers should also appraise the value of the MoCA in a diagnostic workup enabling clinicians to attain relevant outcomes for the SAH patients, such as the benefits of earlier diagnosis. A stepwise protocol including cognitive screening, followed by further assessment with a full neuropsychological evaluation for the patients with abnormal screening test results, would be easy to implement in routine care, guiding clinicians on how to address this complex problem.

After the publication of the international recommendations on the use of MoCA in SAH patients [7], the number of scientific papers reporting the application of MoCA in this type of stroke increased (see Supplementary Material S2). Before 2019, MoCA was used in 23 studies on different aspects of SAH, including research on interventions, the prevalence of cognitive impairment, biomarkers, neuroimaging aspects, driving performance, follow-up, and employment status. In the last two years, 26 studies reported using the MoCA test in SAH patients, including research on interventions, clinical status, prevalence and follow-up, neuroimaging aspects, other biomarkers, and driving ability (see Supplementary Material S1).

Nonetheless, the two studies on the diagnostic accuracy of MoCA were performed before these recommendations. The absence of more recent studies on this topic, and the increasing number of research using MoCA in SAH patients could be explained by the fact that there is a general impression that there is a well-established consensus on the use of MoCA in SAH patients.

We advocate for international recommendations to be evidence-based, with decisions following a well-defined theoretic framework [33]. There are several systems for producing evidence-based recommendations. For example, the Grading of Recommendations Assessment, Development and Evaluation (GRADE) working group proposed a systematic, explicit approach for developing evidence-based guidelines [34] that is now widely used by international expert panels. For diagnostic test accuracy recommendations, the first step would consist of formulating the problem and identifying the important outcomes in terms of population, index test, comparator or reference test, and outcome. The second step comprises gathering the evidence; this should be should be systematic, comprehensive, and reproducible, following the procedures for a systematic review. The last step should evaluate the quality of the evidence, keeping in mind that systematic reviews and meta-analysis are generally considered to be of higher-quality than unfiltered evidence like individual studies [33]. The GRADE system further rates the quality of the evidence found in literature, enabling a systematic and transparent method for evaluating the strength of recommendations [33,34].

The present systematic review identified many research gaps regarding the use of the MoCA in patients with SAH. The main problem is the need for high-quality, cross-sectional studies on the psychometric properties and the optimal cutoff for detecting cognitive impairment in this specific population. Furthermore, it is essential to investigate the role of the MoCA test in different SAH stages, including acute, subacute, and chronic phases. Another subject to be considered for future research is the reliability of MoCA for detecting the changes in cognitive function over time. In addition, future clinical trials should employ both MoCA and extensive neuropsychological testing to document whether the MoCA accurately identifies the cognitive changes after an intervention.

A recent systematic review including 65 studies investigated the cognitive deficits associated with SAH [35]. Nussbaum et al. searched two databases (PubMed and Google Scholar) for studies published between 2010–2019, reporting patients with aneurismal SAH that experienced long-term cognitive deficits. They found that cognitive impairment, including mild forms, was present in 40–70% of SAH survivors. The MoCA and MMSE were the most frequently used to assess cognitive functioning during follow-up periods. The authors concluded that SAH patients, including those that appear normal at the time of hospital discharge, may present cognitive impairment that, although difficult to detect, can interfere with daily functioning. However, the researchers did not assess the quality of the included diagnostic test accuracy studies [35].

Our systematic review has some limitations as we did not perform meta-analyses; the extensive literature search revealed only two diagnostic accuracy studies with relatively small samples of patients. In addition, the included studies presented significant heterogeneity concerning study design, patients’ status (i.e., time from SAH), demographic differences, language and educational background, and reference standards. However, the present systematic review provides an extensive review of the literature, identifying gaps in the knowledge on the use of MoCA in patients with SAH, as the examination and presentation of what has not been investigated or reported generally require an exhaustive investigation of all of that is available. Furthermore, a systematic review may be undertaken to confirm or refute whether or not current practice is based on relevant evidence, to establish the quality of that evidence, and address any uncertainty or variation in practice that may be occurring [36].

## 5. Conclusions

Our comprehensive presentation of the studies that investigated the use of MoCA in SAH patients provides a broad picture of the current state of the knowledge in the field. It identifies the research gaps in this area, including the very low number of diagnostic test accuracy studies and the lack of knowledge around the optimal threshold. Therefore, we delineate areas for future research initiatives. Additionally, we summarized the quality appraisal of the included studies, offering an overview of the validity of the available evidence.

In conclusion, despite the limitations mentioned above, the present work represents the first systematic review of the literature published on the accuracy of MoCA in detecting cognitive impairment in SAH patients, presenting an accurate state of knowledge in this area.

## Figures and Tables

**Table 1:** Characteristics of the included studies.

Study	Country	Study Type	Sample of Patients	Gender (Female/Male)	Age Years	Education (Years +/− SD)	Disease Duration (=/− SD)	MoCA (+/− SD)	MMSE (+/− SD)	Functional Status
Schweizer 2012 [22]	Canada	Cross-sectional	32	19/13	55.2 ± 7.8 (SD)	15.8 ± 3.8	29.3 ± 17.5 months	25.4 ± 2.8	29.3 ± 1.1	Hunt and Hess grade (I/II/III) 2/17/12 WFNS grade (I/II/III/IV) 22/3/3/2 Fisher grade (I/II/III/IV) 2/3/10/15
Wong 2013 [23]	China	Cross-sectional	74	50/24	Median 58 (IQR 49–66)	N/R	2–4 weeks	optimal cutoff: ≤18	optimal cutoff: ≤24	WFNS grade (I/II/III/IV/V) 48/15/4/6/1 mRS 0/1/2/3/4/5 9/4/28/15/17/1
			80	55/25	Median 52 (IQR 47–61)	N/R	1 year	optimal cutoff: ≤22	optimal cutoff: ≤24	WFNS grade (I/II/III/IV/V) 45/21/1/9/4 mRS 0/1/2/3/4/5 22/12/33/11/1/1

## Data Availability

All data included in the review are provided in the tables, the text and the Supplementary Materials.

## Supplementary Materials

The following supporting information can be downloaded at: https://www.mdpi.com/article/10.3390/jcm11164679/s1, Supplementary Material S1: PRISMA DTA checklist. Supplementary Material S2: List of the excluded studies.
