# Supplementary material for: Montreal Cognitive Assessment for Evaluating Cognitive Impairment in Subarachnoid Hemorrhage: A Systematic Review
Source: J Clin Med. 2022 Aug 10;11(16):4679. doi: 10.3390/jcm11164679 (PMC9409824; doi:10.3390/jcm11164679)
Supplement: Supplementary file 1 [file jcm-11-04679-s001.zip › jcm-1827028-supplementary/Supplementary Material S2. List of excluded studies.pdf]

List of excluded studies

1. Ali A, Tanirgan G, Sabanci PA, Sivrikoz N, Abdullah T, Sencer A, Sencer S, Orhan-Sungur M, Akinci IO. Relation of gray-white matter ratio with long-term cognitive functions and quality of life in patients with mild to moderate aneurysmal subarachnoid hemorrhage: a prospective observational study. Acta Neurochir (Wien). 2018;160(1):181-189. doi: 10.1007/s00701-017-3374-y. – neuroimaging
2. Ali A, Bitir B, Abdullah T, Sabanci PA, Aras Y, Aydoseli A, Tanirgan G, Sencer S, Akinci IO. Gray-to-white matter ratio predicts long-term recovery potential of patients with aneurysmal subarachnoid hemorrhage. Neurosurg Rev. 2020;43(1):195-202. doi: 10.1007/s10143-018-1029-y. – neuroimaging
3. Ali A, Abdullah T, Orhan-Sungur M, Orhun G, Aygun E, Aygun E, Sabanci PA, Aras Y, Akinci IO. Transpulmonary thermodilution monitoring-guided hemodynamic management improves cognitive function in patients with aneurysmal subarachnoid hemorrhage: a prospective cohort comparison. Acta Neurochir (Wien). 2019;161(7):1317-1324. doi: 10.1007/s00701-019-03922-4. – intervention and follow-up
4. Badjatia N, Monahan A, Carpenter A, Zimmerman J, Schmidt JM, Claassen J, Connolly ES, Mayer SA, Karmally W, Seres D. Inflammation, negative nitrogen balance, and outcome after aneurysmal subarachnoid hemorrhage. Neurology. 2015;84(7):680-7. doi: 10.1212/WNL.0000000000001259. – not MoCA
5. Badjatia N, Sanchez S, Judd G, Hausladen R, Hering D, Motta M, Parikh G, Chang W, Morris N, Simard JM, Sorkin J, Wittenberg GF, Ryan AS. Neuromuscular Electrical Stimulation and High-Protein Supplementation After Subarachnoid Hemorrhage: A Single-Center Phase 2 Randomized Clinical Trial. Neurocrit Care. 2021;35(1):46-55. doi: 10.1007/s12028-020-01138-4. – intervention
6. Boerboom W, Heijenbrok-Kal MH, van Kooten F, Khajeh L, Ribbers GM. Unmet needs, community integration and employment status four years after subarachnoid haemorrhage. J Rehabil Med. 2016;48(6):529-34. doi: 10.2340/16501977-2096. – follow-up, employment status
7. Brawanski N, Kashefiolasl S, Won SY, Tritt S, Berkefeld J, Senft C, Seifert V, Konczalla J. Does aneurysm side influence the infarction side and patients´ outcome after subarachnoid hemorrhage? PLoS One. 2019;14(11):e0224013. doi: 10.1371/journal.pone.0224013.– not MoCA
8. Bründl E, Proescholdt M, Störr EM, Schödel P, Bele S, Höhne J, Zeman F, Brawanski A, Schebesch KM. Endogenous calcitonin gene-related peptide in cerebrospinal fluid and early quality of life and mental health after good-grade spontaneous subarachnoid hemorrhage-a feasibility series. Neurosurg Rev. 2021;44(3):1479-1492. doi: 10.1007/s10143-020-01333-z. – not MoCA
9. Bründl E, Proescholdt M, Schödel P, Bele S, Höhne J, Zeman F, Stoerr EM, Brawanski A, Schebesch KM. Excessive release of endogenous neuropeptide Y into cerebrospinal fluid after treatment of spontaneous subarachnoid haemorrhage and its possible impact on self-reported neuropsychological performance - results of a prospective clinical pilot study on good-grade patients. Neurol Res. 2018;40(12):1001-1013. doi: 10.1080/01616412.2018.1508547. – not MoCA
10. Buunk AM, Spikman JM, Metzemaekers JDM, van Dijk JMC, Groen RJM. Return to work after subarachnoid hemorrhage: The influence of cognitive deficits. PLoS One. 2019;14(8):e0220972. doi: 10.1371/journal.pone.0220972. – not MoCA
11. Byun E, McCurry SM, Opp M, Liu D, Becker KJ, Thompson HJ. Self-efficacy is associated with better sleep quality and sleep efficiency in adults with subarachnoid hemorrhage. J Clin Neurosci. 2020;73:173-178. doi: 10.1016/j.jocn.2019.12.010. – sleep

12. Byun E, McCurry SM, Kim B, Kwon S, Thompson HJ. Sleep Disturbance and Self-management in Adults With Subarachnoid Hemorrhage: A Qualitative Study. Clin Nurs Res. 2022;31(4):632-638. doi: 10.1177/10547738211064036. – not MoCA
13. Carlson AP, Hänggi D, Wong GK, Etminan N, Mayer SA, Aldrich F, Diringer MN, Schmutzhard E, Faleck HJ, Ng D, Saville BR, Bleck T, Grubb R Jr, Miller M, Suarez JI, Proskin HM, Macdonald RL; NEWTON Investigators. Single-Dose Intraventricular Nimodipine Microparticles Versus Oral Nimodipine for Aneurysmal Subarachnoid Hemorrhage. Stroke. 2020;51(4):1142-1149. doi: 10.1161/STROKEAHA.119.027396. – intervention
14. Chalard K, Szabo V, Pavillard F, Djanikian F, Dargazanli C, Molinari N, Manna F, Costalat V, Chanques G, Perrigault PF. Long-term outcome in patients with aneurysmal subarachnoid hemorrhage requiring mechanical ventilation. PLoS One. 2021;16(3):e0247942. doi: 10.1371/journal.pone.0247942. -not MoCA
15. Chen D, Lai Y, Pang M, Xiang S, Zhou D, Wang Y, Wang J, Yan M. Correlations of cognitive dysfunction with subcortical nuclei volumes and diffusion kurtosis imaging parameters in patients with aneurysmal subarachnoid hemorrhage after surgery. Chinese Journal of Neuromedicine 2021; 20(7): 682-688. – article in Chinese
16. Chu AC, Wong GK, Lam SW, Wong A, Ngai K, Poon WS, Mok V. Cognitive impairment in aneurysmal subarachnoid hemorrhage patients with delayed cerebral infarction: prevalence and pattern. Acta Neurochir Suppl. 2015;120:303-6. doi: 10.1007/978-3-319-04981-6_51. – not MoCA
17. Coulibaly AP, Gartman WT, Swank V, Gomes JA, Ruozhuo L, DeBacker J, Provencio JJ. RAR-Related Orphan Receptor Gamma T (RoRγt)-Related Cytokines Play a Role in Neutrophil Infiltration of the Central Nervous System After Subarachnoid Hemorrhage. Neurocrit Care. 2020;33(1):140-151. doi: 10.1007/s12028-019-00871-9. – not MoCA
18. da Costa L, Dunkley BT, Bethune A, Robertson A, Keller A, Pang EW. Increased Frontal Lobe Activation After Aneurysmal Subarachnoid Hemorrhage. Stroke. 2016;47(10):2503-10. doi: 10.1161/STROKEAHA.116.013786. – magnetoencephalography
19. da Costa BBS, Windlin IC, Koterba E, Yamaki VN, Rabelo NN, Solla DJF, Teixeira MJ, Figueiredo EG. Glibenclamide in aneurysmatic subarachnoid hemorrhage (GASH): study protocol for a randomized controlled trial. Trials. 2019;20(1):413. doi: 10.1186/s13063-019-3517-y. – intervention protocol
20. Danala G, Desai M, Shoukat M, Asif A, Heidari M, Zheng B. Applying quantitative image markers to predict clinical measures after aneurysmal subarachnoid hemorrhage. Progress in Biomedical Optics and Imaging - Proceedings of SPIE 2021. DOI: 10.1117/12.2580976. – neuroimaging
21. Delavaran H, Jönsson AC, Lövkvist H, Iwarsson S, Elmståhl S, Norrving B, Lindgren A. Cognitive function in stroke survivors: A 10-year follow-up study. Acta Neurol Scand. 2017;136(3):187-194. doi: 10.1111/ane.12709. – less than 10 SAH patients
22. Duve K, Mishchenko T, Shkrobot S, et al. Cognitive decline, psychological distress and brain atrophy in recovery and residual periods of aneurysmal subarachnoid hemorrhage. Interdisciplinary Neurosurgery-Advanced Techniques and Case Management 2021; 25. DOI: 10.1016/j.inat.2021.101164 – prevalence
23. Duve KV, Mishchenko TS, Shkrobot SI. The comprehensive evaluation of patients' condition in recovery and residual periods of aneurysmal subarachnoid hemorrhage. Wiad Lek. 2020;73(4):777-781. – clinical features, prevalence
24. Eagles ME, Tso MK, Ayling OGS, Wong JH, MacDonald RL. Unfavorable Outcome After Good Grade Aneurysmal Subarachnoid Hemorrhage: Exploratory Analysis. World Neurosurg. 2020;144:e842-e848. doi: 10.1016/j.wneu.2020.09.079. – not MoCA
25. Eagles ME, Tso MK, Macdonald RL. Cognitive Impairment, Functional Outcome, and Delayed Cerebral Ischemia After Aneurysmal Subarachnoid Hemorrhage. World Neurosurg. 2019: S1878-8750(19)30020-8. doi: 10.1016/j.wneu.2018.12.152. – not MoCA

26. Egeto P, Loch Macdonald R, Ornstein TJ, Schweizer TA. Neuropsychological function after endovascular and neurosurgical treatment of subarachnoid hemorrhage: a systematic review and meta-analysis. J Neurosurg. 2018;128(3):768-776. doi: 10.3171/2016.11.JNS162055. – systematic review, not diagnostic test accuracy
27. English SW, Fergusson D, Chassé M, Turgeon AF, Lauzier F, Griesdale D, Algird A, Kramer A, Tinmouth A, Lum C, Sinclair J, Marshall S, Dowlatshahi D, Boutin A, Pagliarello G, McIntyre LA; Canadian Critical Care Trials Group. Aneurysmal SubArachnoid Hemorrhage-Red Blood Cell Transfusion And Outcome (SAHaRA): a pilot randomised controlled trial protocol. BMJ Open. 2016;6(12):e012623. doi: 10.1136/bmjopen-2016-012623. – not MoCA
28. Esmael A, Belal T, Eltoukhy K. Transcranial Doppler for Early Prediction of Cognitive Impairment after Aneurysmal Subarachnoid Hemorrhage and the Associated Clinical Biomarkers. Stroke Res Treat. 2020; 2020:8874605. doi: 10.1155/2020/8874605. – transcranial Doppler, prevalence
29. Gaastra B, Alexander S, Bakker MK, Bhagat H, Bijlenga P, Blackburn S, Collins MK, Doré S, Griessenauer C, Hendrix P, Hong EP, Hostettler IC, Houlden H, Ilhara K, Jeon JP, Kim BJ, Kumar M, Morel S, Nyquist P, Ren D, Ruigrok YM, Werring D, Galea I, Bulters D, Tapper W. Genome-Wide Association Study of Clinical Outcome After Aneurysmal Subarachnoid Haemorrhage: Protocol. Transl Stroke Res. 2022;13(4):565-576. doi: 10.1007/s12975-021-00978-2. – not MoCA
30. Geraghty JR, Lara-Angulo MN, Spegar M, Reeh J, Testai FD. Severe cognitive impairment in aneurysmal subarachnoid hemorrhage: Predictors and relationship to functional outcome. J Stroke Cerebrovasc Dis. 2020;29(9):105027. doi: 10.1016/j.jstrokecerebrovasdis.2020.105027. – clinical features, prevalence, outcome
31. Gerner ST, Reichl J, Custal C, Brandner S, Eyüpoglu IY, Lücking H, Hölter P, Kallmünzer B, Huttner HB. Long-Term Complications and Influence on Outcome in Patients Surviving Spontaneous Subarachnoid Hemorrhage. Cerebrovasc Dis. 2020;49(3):307-315. doi: 10.1159/000508577. E- not MoCA
32. Gong L, Gu Y, Dong Q, et al. A direct correlation between red blood cell indices and cognitive impairment after Aneurysmal Subarachnoid Hemorrhage (aSAH). Current Neurovascular Research 2019; 16(2): 142-147. DOI: 10.2174/1567202616666190412142718. – clinical, prevalence
33. Green T, Demchuk A, Newcommon N. Aggressive surgical interventions for severe stroke: Impact on quality of life, caregiver burden and family outcomes. Can J Neurosci Nurs. 2015;37(2):15-25. Erratum in: Can J Neurosci Nurs. 2016;38(1):7. Demchuk, Andrew [added]; Newcommon, Nancy [added]. – recovery, prevalence
34. Grill E, Klein AM, Howell K, Arndt M, Bodrozic L, Herzog J, Jox R, Koenig E, Mansmann U, Müller F, Müller T, Nowak D, Schaupp M, Straube A, Bender A. Rationale and design of the prospective German registry of outcome in patients with severe disorders of consciousness after acute brain injury. Arch Phys Med Rehabil. 2013;94(10):1870-6. doi: 10.1016/j.apmr.2012.10.040. – less than 10 patients
35. Hänggi D, Etminan N, Aldrich F, Steiger HJ, Mayer SA, Diringer MN, Hoh BL, Mocco J, Faleck HJ, Macdonald RL; NEWTON Investigators. Randomized, Open-Label, Phase 1/2a Study to Determine the Maximum Tolerated Dose of Intraventricular Sustained Release Nimodipine for Subarachnoid Hemorrhage (NEWTON [Nimodipine Microparticles to Enhance Recovery While Reducing Toxicity After Subarachnoid Hemorrhage]). Stroke. 2017;48(1):145-151. doi: 10.1161/STROKEAHA.116.014250. – no data on MoCA
36. Hänggi D, Etminan N, Macdonald RL, Steiger HJ, Mayer SA, Aldrich F, Diringer MN, Hoh BL, Mocco J, Strange P, Faleck HJ, Miller M. NEWTON: Nimodipine Microparticles to Enhance Recovery While Reducing Toxicity After Subarachnoid Hemorrhage. Neurocrit Care. 2015 Oct;23(2):274-84. doi: 10.1007/s12028-015-0112-2. – intervention protocol
37. Hänggi D, Etminan N, Mayer SA, Aldrich EF, Diringer MN, Schmutzhard E, Faleck HJ, Ng D, Saville BR, Macdonald RL; NEWTON Investigators. Clinical Trial Protocol: Phase 3,

Multicenter, Randomized, Double-Blind, Placebo-Controlled, Parallel-Group, Efficacy, and Safety Study Comparing EG-1962 to Standard of Care Oral Nimodipine in Adults with Aneurysmal Subarachnoid Hemorrhage [NEWTON-2 (Nimodipine Microparticles to Enhance Recovery While Reducing TOxicity After SubarachNoid Hemorrhage)]. Neurocrit Care. 2019 Feb;30(1):88-97. – intervention protocol

38. Hasan TF, Haranhalli N, Mbabuike N, Akinduro OO, Garcia OG, Rush BK, Pedraza O, Tawk RG. Aneurysmal subarachnoid hemorrhage: A pilot study for using longitudinal cognitive and neuropsychological testing for functional outcomes. Clin Neurol Neurosurg. 2020;194:105941. doi: 10.1016/j.clineuro.2020.105941. – prevalence, follow-up
39. Hird MA, Vesely KA, Tasneem T, Saposnik G, Macdonald RL, Schweizer TA. A Case-Control Study Investigating Simulated Driving Errors in Ischemic Stroke and Subarachnoid Hemorrhage. Front Neurol. 2018;9:54. doi: 10.3389/fneur.2018.00054. – driving performance
40. Huenges Wajer IM, Visser-Meily JM, Greebe P, Post MW, Rinkel GJ, van Zandvoort MJ. Restrictions and satisfaction with participation in patients who are ADL-independent after an aneurysmal subarachnoid hemorrhage. Top Stroke Rehabil. 2017;24(2):134-141. doi: 10.1080/10749357.2016.1194557. - not MoCA
41. James RF, Khattar NK, Aljuboori ZS, Page PS, Shao EY, Carter LM, Meyer KS, Daniels MW, Craycroft J, Gaughen JR, Chaudry MI, Rai SN, Everhart DE, Simard JM. Continuous infusion of low-dose unfractionated heparin after aneurysmal subarachnoid hemorrhage: a preliminary study of cognitive outcomes. J Neurosurg. 2018; 11:1-8. doi: 10.3171/2017.11.JNS17894. – intervention and follow-up
42. Joswig H, Korte W, Früh S, Epprecht L, Hildebrandt G, Fournier JY, Stienen MN. Neurodegenerative cerebrospinal fluid biomarkers tau and amyloid beta predict functional, quality of life, and neuropsychological outcomes after aneurysmal subarachnoid hemorrhage. Neurosurg Rev. 2018;41(2):605-614. doi: 10.1007/s10143-017-0900-6. – biomarkers and follow-up
43. Krenzlin H, Wesp D, Schmitt J, Frenz C, Kurz E, Masomi-Bornwasser J, Lotz J, Ringel F, Kerz T, Keric N. Decreased Superoxide Dismutase Concentrations (SOD) in Plasma and CSF and Increased Circulating Total Antioxidant Capacity (TAC) Are Associated with Unfavorable Neurological Outcome after Aneurysmal Subarachnoid Hemorrhage. J Clin Med. 2021;10(6):1188. doi: 10.3390/jcm10061188. – biomarkers and follow-up
44. Ladowski D, Qian W, Kapadia AN, Macdonald RL, Schweizer TA. Effect of aneurysmal subarachnoid hemorrhage on word generation. Behav Neurol 2014:610868. doi: 10.1155/2014/610868. – word generation
45. Lai PMR, Du R. Return to Driving Is a Better Predictor of Patient Outcome Than Return to Work After Aneurysmal Subarachnoid Hemorrhage. World Neurosurg. 2020;144:e285-e295. doi: 10.1016/j.wneu.2020.08.113. – not MoCA
46. Li JJ, Lu H. The influence of different interventional embolization techniques on the cognitive function of patients with ruptured anterior communicating artery aneurysm. Journal of Interventional Radiology (China) 2016 Vol. 25 Issue 5 Pages 374-376. DOI: 10.3969/j.issn.1008-794X.2016.05.002. – in Chinese
47. Liao X, Zuo L, Pan Y, Xiang X, Meng X, Li H, Zhao X, Wang Y, Shi J, Wang Y. Screening for cognitive impairment with the montreal cognitive assessment at six months after stroke and transient ischemic attack. Neurol Res. 2021;43(1):15-21. doi: 10.1080/01616412.2020.1819070. – not SAH.
48. Liu J, Song J, Zhao D, et al. Risk factors responsible for the volume of hemorrhage in aneurysmal subarachnoid hemorrhage. Neurology India 2016 Vol. 64 Issue 4 Pages 686-691. DOI: 10.4103/0028-3886.185398. – not MoCA
49. Lukyanchikov VA, Shetova IM, Shtadler VD, et al. Results of revascularizing operations performed in the acute period of subarachnoid hemorrhage. Russian Journal of Neurosurgery

2021 Vol. 23 Issue 2 Pages 14-24. DOI: 10.17650/1683-3295-2021-23-2-14-24– article in Russian

50. Ma J, Yang X, Yin H, Wang Y, Chen H, Liu C, Han G, Gao F. Effect of thyroid hormone replacement therapy on cognition in long-term survivors of aneurysmal subarachnoid hemorrhage. Exp Ther Med. 2015;10(1):369-373. doi: 10.3892/etm.2015.2475. – intervention.
51. Ouellet MA, Rochette A, Miéville C, Poissant L. Portrait of driving practice following a mild stroke: a secondary analysis of a chart audit. Top Stroke Rehabil. 2020;27(3):181-189. doi: 10.1080/10749357.2019.1684049. – driving
52. Paľa A, Schick J, Klein M, Mayer B, Schmitz B, Wirtz CR, König R, Kapapa T. The influence of nimodipine and vasopressors on outcome in patients with delayed cerebral ischemia after spontaneous subarachnoid hemorrhage. J Neurosurg. 2019;132(4):1096-1104. doi: 10.3171/2018.11.JNS182891. – not MoCA
53. Persson HC, Törnbom M, Winsö O, Sunnerhagen KS. Symptoms and consequences of subarachnoid haemorrhage after 7 years. Acta Neurol Scand. 2019;140(6):429-434. doi: 10.1111/ane.13163. – prevalence, follow-up
54. Premat K, Azuar C, Galanaud D, Jacquens A, Dormont D, Degos V, Clarençon F; ACOM Study Group. Pathomechanisms behind cognitive disorders following ruptured anterior communicating aneurysms: A diffusion tensor imaging study. J Neuroradiol. 2022;49(2):187-192. doi: 10.1016/j.neurad.2021.09.005. – not MoCA
55. Rautalin IM, Sebök M, Germans MR, Korja M, Dannecker N, Zindel-Geisseler O, Brugger P, Regli L, Stienen MN. Screening tools for early neuropsychological impairment after aneurysmal subarachnoid hemorrhage. Neurol Sci. 2020;41(4):817-824. doi: 10.1007/s10072-019-04159-w. – correlation with mRS
56. Ray B, Ross SR, Danala G, Aghaei F, Nouh CD, Ford L, Hollabaugh KM, Karfonta BN, Santucci JA, Cornwell BO, Bohnstedt BN, Zheng B, Dale GL, Prodan CI. Systemic response of coated-platelet and peripheral blood inflammatory cell indices after aneurysmal subarachnoid hemorrhage and long-term clinical outcome. J Crit Care. 2019;52:1-9. doi: 10.1016/j.jcrc.2019.03.003. – biomarkers
57. Ray B, Pandav VM, Mathews EA, Thompson DM, Ford L, Yearout LK, Bohnstedt BN, Chaudhary S, Dale GL, Prodan CI. Coated-Platelet Trends Predict Short-Term Clinical OutcomeAfter Subarachnoid Hemorrhage. Transl Stroke Res. 2018;9(5):459-470. doi: 10.1007/s12975-017-0594-7.- biomarkers.
58. Samseethong T, Suansanae T, Veerasarn K, Liengudom A, Suthisisang C. Impact of Early Versus Late Intravenous Followed by Oral Nimodipine Treatment on the Occurrence of Delayed Cerebral Ischemia Among Patients With Aneurysm Subarachnoid Hemorrhage. Ann Pharmacother. 2018;52(11):1061-1069. doi: 10.1177/1060028018778751. – not MoCA
59. Schupper AJ, Eagles ME, Neifert SN, Mocco J, Macdonald RL. Lessons from the CONSCIOUS-1 Study. J Clin Med. 2020;9(9):2970. doi: 10.3390/jcm9092970. – opinion
60. Sharma N, Wig J, Mahajan S, Chauhan R, Mohanty M, Bhagat H. Comparison of postoperative cognitive dysfunction with the use of propofol versus desflurane in patients undergoing surgery for clipping of aneurysm after subarachnoid hemorrhage. Surg Neurol Int. 2020;11:174. doi: 10.25259/SNI_70_2020. – intervention
61. Shen Y, Dong Z, Pan P, Shi H, Song Y. Risk Factors for Mild Cognitive Impairment in Patients with Aneurysmal Subarachnoid Hemorrhage Treated with Endovascular Coiling. World Neurosurg. 2018;119:e527-e533. doi: 10.1016/j.wneu.2018.07.196.– prevalence, follow-up
62. Sousa L, Antunes A, Mendes T, Reimão S, Neto LL, Campos J. Long-term Neuropsychiatric and Neuropsychological Sequelae of Endovascularly Treated Aneurysmal Subarachnoid Hemorrhage. Acta Med Port. 2019;32(11):706-713. doi: 10.20344/amp.10894. – prevalence
63. Stienen MN, Fung C, Bijlenga P, Zumofen DW, Maduri R, Robert T, Seule MA, Marbacher S, Geisseler O, Brugger P, Gutbrod K, Chicherio C, Monsch AU, Beaud V, Rossi S, Früh S, Schmid N, Smoll NR, Keller E, Regli L; MoCA-DCI study group. Measuring the Impact of Delayed

Cerebral Ischemia on Neuropsychological Outcome After Aneurysmal Subarachnoid Hemorrhage-Protocol of a Swiss Nationwide Observational Study (MoCA-DCI Study). Neurosurgery. 2019;84(5):1124-1132. doi: 10.1093/neuros/nyy155. - protocol.

64. Stienen MN, Smoll NR, Weisshaupt R, Fandino J, Hildebrandt G, Studerus-Germann A, Schatlo B. Delayed cerebral ischemia predicts neurocognitive impairment following aneurysmal subarachnoid hemorrhage. World Neurosurg. 2014;82(5):e599-605. doi: 10.1016/j.wneu.2014.05.011. -not MocA
65. Wallmark S, Ronne-Engström E, Lundström E. Predicting return to work after subarachnoid hemorrhage using the Montreal Cognitive Assessment (MoCA). Acta Neurochir (Wien). 2016;158(2):233-9. doi: 10.1007/s00701-015-2665-4. Return to work
66. Wallmark S, Lundström E, Wikström J, Ronne-Engström E. Attention deficits after aneurysmal subarachnoid hemorrhage measured using the test of variables of attention. Stroke. 2015;46(5):1374-6. doi: 10.1161/STROKEAHA.115.009092. MoCA is the reference standard
67. Walter J, Grutza M, Vogt L, Unterberg A, Zweckberger K. The neuropsychological assessment battery (NAB) is a valuable tool for evaluating neuropsychological outcome after aneurysmatic subarachnoid hemorrhage. BMC Neurol. 2020;20(1):429. doi: 10.1186/s12883-020-02003-9. – prevalence, follow-up
68. Western E, Nordenmark TH, Sorteberg W, Karic T, Sorteberg A. Fatigue After Aneurysmal Subarachnoid Hemorrhage: Clinical Characteristics and Associated Factors in Patients With Good Outcome. Front Behav Neurosci. 2021;15:633616. doi: 10.3389/fnbeh.2021.633616. – not MoCA
69. Wong GK, Lam SW, Chan SS, Lai M, Tse PP, Mok V, Poon WS, Wong A. Neuropsychiatric disturbance after aneurysmal subarachnoid hemorrhage. J Clin Neurosci. 2014;21(10):1695-8. doi: 10.1016/j.jocn.2014.02.018. – mRS, IADL, follow-up
70. Wong GK, Lee A, Wong A, Ho FL, Leung SL, Zee BC, Poon WS, Siu DY, Abrigo JM, Mok VC. Clinically important difference of Stroke-Specific Quality of Life Scale for aneurysmal subarachnoid hemorrhage. J Clin Neurosci. 2016;33:209-212. doi: 10.1016/j.jocn.2016.05.029.– not MoCA
71. Wong GK, Lam SW, Wong A, Ngai K, Mok V, Poon WS. Early Cognitive Domain Deficits in Patients with Aneurysmal Subarachnoid Hemorrhage Correlate with Functional Status. Acta Neurochir Suppl. 2016;122:129-32. doi: 10.1007/978-3-319-22533-3_26. – not MoCA
72. Wong GK, Lam S, Ngai K, Wong A, Mok V, Poon WS; Cognitive Dysfunction after Aneurysmal Subarachnoid Haemorrhage Investigators. Evaluation of cognitive impairment by the Montreal cognitive assessment in patients with aneurysmal subarachnoid haemorrhage: prevalence, risk factors and correlations with 3 month outcomes. J Neurol Neurosurg Psychiatry. 2012;83(11):1112-7. doi: 10.1136/jnnp-2012-302217. – prevalence, follow-up
73. Wong GK, Lam SW, Ngai K, Wong A, Poon WS, Mok V. Development of a short form of Stroke-Specific Quality of Life Scale for patients after aneurysmal subarachnoid hemorrhage. J Neurol Sci. 2013;335(1-2):204-9. doi: 10.1016/j.jns.2013.09.033. – Quality of life
74. Wong GK, Lam SW, Ngai K, Wong A, Mok V, Poon WS. Quality of Life after Brain Injury (QOLIBRI) Overall Scale for patients after aneurysmal subarachnoid hemorrhage. J Clin Neurosci. 2014 Jun;21(6):954-6. doi: 10.1016/j.jocn.2013.09.010. – quality of life
75. Wong GK, Lam SW, Wong A, Lai M, Siu D, Poon WS, Mok V. MoCA-assessed cognitive function and excellent outcome after aneurysmal subarachnoid hemorrhage at 1 year. Eur J Neurol. 2014 May;21(5):725-30. doi: 10.1111/ene.12363. – mRS, IADL, follow-up
76. Wong GK, Nung RC, Sitt JC, Mok VC, Wong A, Ho FL, Poon WS, Wang D, Abrigo J, Siu DY. Location, Infarct Load, and 3-Month Outcomes of Delayed Cerebral Infarction After Aneurysmal Subarachnoid Hemorrhage. Stroke. 2015;46(11):3099-104. doi: 10.1161/STROKEAHA.115.010844. – DCI
77. Wong GK, Chan DY, Siu DY, Zee BC, Poon WS, Chan MT, Gin T, Leung M; HDS-SAH Investigators. High-dose simvastatin for aneurysmal subarachnoid hemorrhage: multicenter

randomized controlled double-blinded clinical trial. Stroke. 2015;46(2):382-8. doi: 10.1161/STROKEAHA.114.007006. – intervention
78. Wong GK, Wong A, Zee BC, Poon WS, Chan MT, Gin T, Siu DY, Mok VC. Cognitive outcome in acute simvastatin treatment for aneurysmal subarachnoid hemorrhage: A propensity matched analysis. J Neurol Sci. 2015;358(1-2):58-61. doi: 10.1016/j.jns.2015.08.013. - intervention
79. Woo PYM, Ho JWK, Ko NMW, Li RPT, Jian L, Chu ACH, Kwan MCL, Chan Y, Wong AKS, Wong HT, Chan KY, Kwok JCK. Randomized, placebo-controlled, double-blind, pilot trial to investigate safety and efficacy of Cerebrolysin in patients with aneurysmal subarachnoid hemorrhage. BMC Neurol. 2020;20(1):401. doi: 10.1186/s12883-020-01908-9. – intervention
80. Ye ZN, Cai MQ, Jin Y, Chen M. The influence of lateral ventricle puncture and drainage on the quality of life and prognoses of patients with aneurysmal subarachnoid hemorrhage. International Journal of Clinical and Experimental Medicine 2020; 13(4): 2259-2267. - intervention
81. Zhang LL and Mou L. Investigation of cognitive function and analysis of related factors in patients with aneurysmal subarachnoid hemorrhage after receiving interventional treatment. Journal of Interventional Radiology (China) 2015 Vol. 24 Issue 8 Pages 730-732. DOI: 10.3969/j.issn.1008-794X.2015.08.020 – article in Chinese
82. Zhou D, Wei D, Xing W, Li T, Huang Y. Effects of craniotomy clipping and interventional embolization on treatment efficacy, cognitive function and recovery of patients complicated with subarachnoid hemorrhage. American Journal of Translational Research 2021; 13(5): 5117-5126. – intervention
83. Zhou XM, Xu Y, Jiang JF, Zhou WC. Time-related intervention efficacy, serum ferritin levels, and prognosis in patients with aneurismal subarachnoid hemorrhage. International Journal of Clinical and Experimental Medicine 2020; 13(11): 8411-8419 – intervention
84. Zweifel-Zehnder AE, Stienen MN, Chicherio C, Studerus-Germann A, Bläsi S, Rossi S, Gutbrod K, Schmid N, Beaud V, Mondadori C, Brugger P, Sacco L, Müri R, Hildebrandt G, Fournier JY, Keller E, Regli L, Fandino J, Mariani L, Raabe A, Daniel RT, Reinert M, Robert T, Schatlo B, Bijlenga P, Schaller K, Monsch AU; Swiss SOS study group. Call for uniform neuropsychological assessment after aneurysmal subarachnoid hemorrhage: Swiss recommendations. Acta Neurochir (Wien). 2015;157(9):1449-58. doi: 10.1007/s00701-015-2480-y. – opinion paper
